# Merkel cell polyomavirus DNA in CSF of paediatric patients with suspected meningitis or encephalitis

**DOI:** 10.1186/s12985-026-03261-8

**Published:** 2026-07-25

**Authors:** Negar Hemmati, Fatemeh Hoda Fallah, Bahman Abedi Kiasari

**Affiliations:** 1 Department of Microbiology and Immunology, University of Tehran, Tehran, Iran; 2 Valiasr Hospital, Imam Khomeini Hospital, Tehran University of Medical Sciences (TUMS), Tehran, Iran

**Keywords:** Human polyomavirus, Merkel cell polyomavirus (MCPyV), Encephalitis, Meningitis, PCR, CSF

## Abstract

**Background:**

The clinical significance of Merkel cell polyomavirus (MCPyV) in pediatric central nervous system (CNS) infections remains uncertain, particularly in immunocompromised children.

**Objective:**

To determine the frequency of MCPyV DNA in cerebrospinal fluid (CSF) from children with suspected viral meningitis or encephalitis and evaluate its association with HIV infection, CSF findings, and clinical outcomes.

**Methods:**

CSF samples from 46 pediatric patients with suspected viral CNS infection were analyzed using TaqMan probe-based quantitative PCR targeting the large T-antigen gene. Positive specimens were confirmed by amplification of an independent VP1 target and bidirectional Sanger sequencing following conventional PCR re-amplification. Clinical, laboratory, and outcome data were compared using appropriate statistical tests.

**Results:**

MCPyV DNA was detected in 6/46 patients (13.0%), all of whom were HIV-positive (*p* = 0.001; sensitivity analysis excluding patients with unknown HIV status, *p* = 0.002). Four isolates belonged to genotype I and two to genotype IIc. Compared with MCPyV-negative patients, MCPyV-positive patients had higher CSF lymphocyte percentages (89% vs. 84%; *p* = 0.048), higher protein concentrations (79 vs. 68 mg/dL; *p* = 0.022), lower glucose concentrations (51 vs. 55 mg/dL; *p* = 0.038), and more frequent altered consciousness (83.3% vs. 37.5%; *p* = 0.039). EBV DNA co-detection was identified in one MCPyV-positive patient.

**Conclusions:**

MCPyV DNA was detected exclusively in HIV-positive children and was associated with inflammatory CSF abnormalities. These findings suggest a possible association between MCPyV detection, immunosuppression, and CNS inflammation but do not establish causality. Larger multicenter studies are required.

## Introduction

Human polyomaviruses (HPyVs) are a family of small, non-enveloped, double-stranded DNA viruses that establish widespread, asymptomatic, and lifelong persistent infections in the human population [1–3]. Following primary infection, typically in early childhood, these viruses establish latency in various tissues, including the kidneys, lymphoid organs, and the central nervous system (CNS) [4, 5]. Under conditions of significant immunosuppression, reactivation can occur, leading to a spectrum of clinical diseases. The most well-characterized examples are JC polyomavirus (JCPyV), the etiological agent of the fatal demyelinating disease progressive multifocal leukoencephalopathy (PML), and BK polyomavirus (BKPyV), associated with nephropathy in transplant recipients and hemorrhagic cystitis [6–8].

In 2008, using a novel digital transcriptome subtraction technique, Feng and colleagues identified a previously unknown polyomavirus in Merkel cell carcinoma (MCC), an aggressive neuroendocrine skin cancer [9, 10]. This virus was named Merkel cell polyomavirus (MCPyV). Subsequent studies have robustly demonstrated that MCPyV is clonally integrated into the genome of approximately 80% of MCC tumors, providing strong evidence for its direct oncogenic role [11–14]. The virus encodes two primary oncoproteins, the large T-antigen (LT-ag) and small T-antigen (sT-ag) [15, 16]. In MCC, LT-ag is typically expressed in a truncated form, whereas sT-ag is generally expressed as a full-length, functionally intact protein; this distinction is critical for understanding viral oncogenesis and the rationale for PCR-based detection [10, 17, 18]. In this study, we targeted the LT-ag and VP1 regions of the MCPyV genome to assess viral presence in cerebrospinal fluid.

Although the link between MCPyV and cutaneous malignancy is well-established, its potential tropism for the CNS and role in neurological diseases remain poorly understood [19–21]. MCPyV DNA has been detected in various sites beyond the skin [22, 23], including the respiratory tract [24–26], blood [27], and present even in urban sewage [28], suggesting multiple potential routes of transmission and dissemination. Importantly, several studies have now reported the detection of MCPyV DNA in cerebrospinal fluid (CSF) specimens from patients with diverse neurological conditions, although its clinical significance remains a subject of debate [1, 20, 21, 29]. Whether the presence of MCPyV in the CSF represents an active neuroinvasive infection, a latent resident of the CNS, or an incidental finding from peripheral viral shedding is unclear. This question is particularly relevant in immunocompromised children, such as those with HIV infection, who may be predisposed to opportunistic CNS infections. Understanding this is particularly critical, as primary MCPyV infection is acquired in early childhood, and seroprevalence increases with age, reaching 60–80% in healthy adults, mirroring the epidemiology of other neurotropic polyomaviruses [30, 31].

Given the expanding use of immunosuppressive therapies and the persistent burden of HIV-associated immunodeficiency, there is a crucial need to fully characterize the clinical spectrum of all HPyVs [32–35]. In pediatric populations, where CNS infections carry a high risk of morbidity and mortality, and a substantial proportion of cases remain etiologically unexplained, the role of emerging pathogens like MCPyV warrants focused investigation [36, 37]. Therefore, the aim of this study was to determine the frequency of MCPyV DNA in the CSF of pediatric patients, particularly focusing on HIV-positive immunocompromised children, and to explore its potential association with CNS disease.

## Materials and methods

### Study populations and samples

A total of 46 CSF specimens were collected consecutively from pediatric patients who underwent lumbar puncture for suspected viral encephalitis or meningoencephalitis between April and December 2024 at the Clinical Virology Laboratory, Tehran, Iran. Children were eligible for inclusion if they were aged between 6 months and 14 years and presented with acute neurological manifestations, including fever with headache, neck stiffness, altered level of consciousness, seizures, or focal neurological deficits. These symptoms were required to be accompanied by CSF profiles consistent with non-bacterial meningitis, defined as lymphocytic pleocytosis (≥ 5 WBC/µL with > 50% lymphocytes), normal or mildly reduced glucose concentrations, and normal to moderately elevated protein levels [22]. Patients were excluded if routine microbiological investigations (Gram staining and bacterial culture) confirmed bacterial or fungal CNS infection, or if an alternative non-infectious neurological diagnosis was established.

All samples were obtained during the acute phase of illness, immediately transported to the laboratory, and stored at − 70 °C until analysis. CSF analysis consistently demonstrated leukocyte counts below 1000 cells/mL, negative Gram staining, and no bacterial growth after five days of incubation. CSF specimens were aliquoted into multiple vials to prevent repeated freeze-thaw cycles, and all laboratory procedures were performed under Class II biosafety conditions.

HIV serostatus was available for 27 of the 46 patients (58.7%). The HIV status of the remaining patients was unknown because of incomplete medical records or lack of parental consent. Immunologic parameters collected for the HIV-positive children included antiretroviral therapy (ART), absolute CD4 + cell count, absolute CD8 + cell count, HIV viral load, and WHO AIDS staging.

All CSF samples were systematically screened for a panel of common viral co-pathogens, including herpes simplex virus (HSV-1/2), Varicella zoster virus (VZV), Epstein-Barr virus (EBV), Human herpesvirus 6 (HHV-6), and enteroviruses, using validated PCR assays in the laboratory. Each assay included both positive and negative controls, and laboratory personnel were blinded to patient clinical status. Only one patient tested positive for EBV; all other co-pathogens were negative.

Demographic information and relevant clinical features, including age, sex, fever, seizure, altered consciousness, focal neurological deficits, and prior medical history, were recorded using standardized case report forms. Clinical data were entered into a secure, access-controlled electronic database.

Written informed consent was obtained from the parents or legal guardians of all participants. The study protocol was approved by the institutional ethics committee, and all procedures were conducted in accordance with the Declaration of Helsinki. All specimens were anonymized by removing personal identifiers and assigning unique research codes prior to laboratory processing. Laboratory staff performing molecular analyses were blinded to the clinical and HIV status of the patients to minimize potential bias.

### Molecular analysis

Viral DNA was extracted from 200 µL of each CSF specimen using the QIAamp^®^ MinElute^®^ Virus Spin Kit (Qiagen, Hilden, Germany) according to the manufacturer’s instructions under Class II biosafety conditions. All specimens were aliquoted before extraction to avoid repeated freeze–thaw cycles and were processed by laboratory personnel blinded to the patients’ clinical characteristics and HIV status.

MCPyV DNA was detected using TaqMan probe-based quantitative real-time PCR (qPCR) assays. No SYBR Green-based assay was used for MCPyV detection in this study. Primer and probe sequences targeting the large T-antigen (LT-ag) and VP1 genes were adopted from previously published assays, with modifications to the reaction conditions (Table 1). The LT-ag assay served as the primary screening assay, whereas the VP1 assay was used as an independent confirmatory target.

Each qPCR reaction was performed in a final volume of 25 µL containing 12.5 µL of 2× TaqMan Universal PCR Master Mix (Applied Biosystems, Foster City, CA, USA), 0.4 µM of each primer, 0.2 µM of the corresponding TaqMan probe, 5 µL of extracted DNA, and nuclease-free water.

Thermal cycling consisted of an initial denaturation at 95 °C for 10 min, followed by 45 cycles of denaturation at 95 °C for 15 s and annealing/extension at 60 °C for 60 s. Samples were considered MCPyV-positive when amplification was detected with a cycle threshold (Ct) value ≤ 38 in the LT-ag assay and subsequently confirmed by amplification of the independent VP1 target. Samples positive in only one assay were retested, and only specimens repeatedly positive in the LT-ag assay and confirmed by VP1 amplification were classified as MCPyV-positive.

Standard curves generated from ten-fold serial dilutions of plasmid DNA containing the MCPyV target sequence (10⁶–10¹ copies/reaction) were used only for analytical validation of assay performance. Ct values were recorded for all positive samples and were used as indicators of MCPyV DNA burden. Amplification efficiency ranged from 92% to 98%, with correlation coefficients (R²) ≥ 0.99 for all assays.

Positive controls consisted of MCPyV-positive Merkel cell carcinoma cell lysate, whereas negative controls included nuclease-free water, extraction blank controls, and CSF specimens obtained from laboratory-negative pediatric controls. Inter- and intra-assay variability was monitored using replicate control samples in each run.

All CSF specimens were also systematically screened for common viral CNS pathogens, including herpes simplex virus type 1 and 2 (HSV-1/2), varicella-zoster virus (VZV), Epstein–Barr virus DNA (EBV DNA), human herpesvirus 6 (HHV-6), and enteroviruses using validated qPCR assays. Only one patient tested positive for EBV DNA, whereas all other viral co-pathogens were negative.

To confirm MCPyV detection, all qPCR-positive specimens underwent bidirectional Sanger sequencing following re-amplification by a conventional PCR assay targeting the LT-ag region. The resulting PCR amplicons were purified using a commercial PCR purification kit and sequenced using the BigDye Terminator Cycle Sequencing Kit (Applied Biosystems, Foster City, CA, USA) on an ABI 3100 Genetic Analyzer. Sequence identity was confirmed by comparison with reference MCPyV sequences available in the GenBank database using the BLASTn algorithm.

**Table 1:** Primer and probe sequences used for TaqMan probe-based quantitative real-time PCR (qPCR) detection of Merkel cell polyomavirus (MCPyV) DNA in cerebrospinal fluid (CSF) specimens

Primer/Probe	Target Gene	Sequence (5′→3′)	Amplicon Size (bp)	Reference
LT.1 F	Large T-antigen (LT-ag)	CCACAGCCAGAGCTCTTCCT	146	[27]
LT.1R	Large T-antigen (LT-ag)	TGGTGGTCTCCTCTCTGCTACTG		[27]
LT.1 Probe	Large T-antigen (LT-ag)	FAM-TCCTTCTCAGCGTCCCAGGCTTCA-TAMRA	—	[27]
VP1 Forward	VP1	TGCCTCCCACATCTGCAAT	59	[38]
VP1 Reverse	VP1	GTGTCTCTGCCAATGCTAAATGA		[38]
VP1 Probe	VP1	6FAM-TGTCACAGGTAATATC-MGBNFQ	—	[38]

The LT-ag assay was used as the primary screening assay, whereas the VP1 assay served as an independent confirmatory target

CSF, cerebrospinal fluid; LT-ag, large T-antigen; MCPyV, Merkel cell polyomavirus; qPCR, quantitative real-time polymerase chain reaction; VP1, viral capsid protein 1

### Sequencing and phylogenetic analysis

All MCPyV-positive samples identified by TaqMan probe-based qPCR were subjected to confirmatory sequence analysis. Sequencing was not performed directly on the qPCR products. Instead, MCPyV-positive DNA extracts were re-amplified by a separate conventional PCR assay targeting the LT-ag region using the same LT-ag primer pair employed in the TaqMan qPCR assay, but without the TaqMan probe.

Conventional PCR was performed in a final volume of 25 µL containing 12.5 µL of 2× PCR Master Mix, 0.4 µM of each LT-ag primer, 5 µL of extracted DNA, and nuclease-free water. Cycling conditions consisted of an initial denaturation at 95 °C for 10 min, followed by 40 cycles of denaturation at 95 °C for 15 s, annealing at 60 °C for 60 s, and a final extension at 72 °C for 7 min.

The resulting LT-ag PCR amplicons were purified using a commercial PCR purification kit and subjected to bidirectional Sanger sequencing using the BigDye Terminator Cycle Sequencing Kit (Applied Biosystems, Foster City, CA, USA) on an ABI 3100 Genetic Analyzer (Applied Biosystems).

Raw chromatograms were inspected manually for sequence quality, and consensus sequences were assembled and edited using Sequencher software (version 4.6; Gene Codes Corp., Ann Arbor, MI, USA). Viral identity was confirmed by comparison of the obtained sequences with reference MCPyV sequences deposited in the GenBank database using the BLASTn algorithm of the National Center for Biotechnology Information (NCBI). Genotype assignment was based on sequence similarity and phylogenetic clustering with previously characterized MCPyV reference strains, including Type I, Type II, and Type IIc genotypes.

For phylogenetic reconstruction, confirmed LT-ag sequences were aligned with representative MCPyV reference strains using the ClustalW algorithm implemented in MEGA software (version 7.0). Reference sequences included representative Type I (HQ696518), Type II (HQ696519), and Type IIc (JQ958895) strains retrieved from GenBank. Phylogenetic trees were constructed using the neighbor-joining method with the Kimura two-parameter substitution model. The robustness of the inferred tree topology was evaluated by bootstrap analysis with 1,000 replicates. Final phylogenetic trees were visualized and annotated using FigTree software (version 1.4.4).

### Clinical outcomes

Clinical outcomes were assessed at hospital discharge and during a standardized 1-month follow-up visit. Full recovery was defined as complete resolution of all neurological symptoms without any residual deficits. Partial recovery was defined as the presence of one or more of the following persistent clinical manifestations at the 1-month assessment: (i) neurological deficits, including motor or sensory impairment; (ii) residual seizures; (iii) cognitive or developmental impairment identified by age-appropriate neurological examination; or (iv) prolonged hospitalization exceeding 14 days.

Patients classified as having partial recovery at the 1-month assessment continued to receive clinical follow-up by paediatric neurologists as part of routine medical care. Available follow-up information beyond the predefined 1-month study endpoint was extracted retrospectively from the medical records. Because the timing and duration of these subsequent clinical evaluations were determined by routine patient care rather than a predefined study protocol, post-study follow-up data were considered descriptive and were not included in the formal statistical analyses.

For comparative analyses, two groups were defined: HIV-positive/MCPyV-positive (HIV+/MCPyV+, *n* = 6) and HIV-positive/MCPyV-negative (HIV+/MCPyV−, *n* = 11). The following demographic, clinical, and laboratory variables were systematically recorded and compared between groups: age, sex, CSF white blood cell count, protein concentration, glucose concentration, fever, seizures, ICU admission, mortality, and clinical outcome (full versus partial recovery). All clinical evaluations were performed by pediatric neurologists blinded to MCPyV status. Data were entered into a secure, access-controlled electronic database, and all patient identifiers were anonymized before analysis.

### Statistical analysis

All statistical analyses were performed using IBM SPSS Statistics for Windows, version 26.0 (IBM Corp., Armonk, NY, USA). Continuous variables with normal distribution were compared using independent-samples t-test, whereas non-normally distributed variables, including CSF WBC count, protein concentration, and glucose concentration, were compared using the Mann–Whitney U test following Shapiro–Wilk normality testing. Categorical variables, including fever, seizures, ICU admission, mortality, and clinical outcomes, were compared using Fisher’s exact test or chi-square test, as appropriate. Correlations between MCPyV DNA burden and clinical or CSF parameters were assessed using Spearman’s rank correlation. MCPyV DNA burden was evaluated using Ct values, with lower Ct values indicating higher MCPyV DNA burden. A p-value < 0.05 was considered statistically significant. All analyses were performed on anonymized datasets, and all laboratory and clinical personnel were blinded to group assignments to reduce bias. Additionally, a group of laboratory-negative CSF controls (*n* = 10) was included in all qPCR runs to ensure assay specificity and validate clinical associations.

## Results

### Demographic and clinical characteristics of the study population

A total of 46 pediatric patients with suspected viral encephalitis or meningoencephalitis were included in this study. The mean age of the cohort was 6.4 ± 3.8 years (range: 0.6–14 years), with an equal distribution of males and females (23/46, 50.0% each). HIV serostatus was positive in 17 patients (37.0%), negative in 15 (32.6%), and unknown in 14 (30.4%) because of incomplete medical records or lack of parental consent. All HIV-positive patients had moderate-to-severe immunosuppression according to WHO clinical staging and were receiving standard triple antiretroviral therapy. Immunological evaluation demonstrated absolute CD4 + cell counts ranging from 120 to 250 cells/µL, CD8 + cell counts from 500 to 800 cells/µL, and HIV viral loads between 50,000 and 150,000 copies/mL.

The clinical diagnoses included meningitis in 20 patients (43.5%), encephalitis in 13 (28.3%), and meningoencephalitis in 13 (28.3%). Fever was present in all patients (46/46, 100%), whereas seizures, altered consciousness, and neck stiffness were observed in 16 (34.8%), 20 (43.5%), and 28 (60.9%) patients, respectively.

CSF analysis demonstrated a median white blood cell count of 85 cells/µL (IQR: 62–120), with a median lymphocyte proportion of 85% (IQR: 82–88). The median CSF protein concentration was 70 mg/dL (IQR: 62–85), whereas the median CSF glucose concentration was 54 mg/dL (IQR: 50–58). The baseline demographic, clinical, and laboratory characteristics of the study population are summarized in Table 2. Comparative analyses between HIV+/MCPyV + and HIV+/MCPyV− patients are presented in the subsequent sections.

**Table 2:** Baseline demographic, clinical, and laboratory characteristics of the pediatric study population (*N* = 46)

Parameter	Value
Age (years), mean ± SD	6.4 ± 3.8
Gender, n (%)	
Male	23 (50.0)
Female	23 (50.0)
HIV Status, n (%)	
Positive	17 (37.0)
Negative	15 (32.6)
Unknown	14 (30.4)
Clinical Diagnosis, n (%)	
Meningitis	20 (43.5)
Encephalitis	13 (28.3)
Meningoencephalitis	13 (28.3)
CSF Parameters, median (IQR)	
WBC (cells/µL)	85 (62–120)
Lymphocytes (%)	85 (82–88)
Protein (mg/dL)	70 (62–85)
Glucose (mg/dL)	54 (50–58)
Clinical Signs, n (%)	
Fever	46 (100)
Seizure	16 (34.8)
Altered consciousness	20 (43.5)
Neck stiffness	28 (60.9)

Continuous variables are presented as mean ± standard deviation (SD) or median (interquartile range [IQR]), whereas categorical variables are presented as number (%)

CSF, cerebrospinal fluid; HIV, human immunodeficiency virus; IQR, interquartile range; SD, standard deviation; WBC, white blood cell count

### Clinical outcomes and longitudinal follow-up

At the predefined 1-month follow-up assessment, 32 patients (69.6%) achieved full recovery, whereas 14 (30.4%) met the predefined criteria for partial recovery. No deaths occurred during the study period. Clinical outcomes and longitudinal follow-up findings are summarized in Table 3.

**Table 3:** Clinical outcomes at the predefined 1-month assessment and longitudinal neurological follow-up of patients with partial recovery

Variable	*n* (%)	Follow-up findings
Clinical outcome at 1-month assessment		
Full recovery	32 (69.6)	Complete resolution of neurological symptoms
Partial recovery	14 (30.4)	Additional neurological follow-up available for all patients
Death	0 (0)	None
Residual neurological manifestations among patients with partial recovery (*n* = 14)		
Residual seizures	3 (21.4)	Progressive reduction in seizure frequency; complete seizure control achieved in one patient
Mild motor deficits (including hemiparesis)	2 (14.3)	Significant motor recovery; only minimal residual weakness at latest evaluation
Cognitive impairment	2 (14.3)	Progressive improvement during serial neurological assessments
Developmental delay/behavioral abnormalities	2 (14.3)	Gradual neurological and functional improvement; continued monitoring when clinically indicated
Persistent headache/concentration difficulties	3 (21.4)	Symptoms resolved completely or markedly improved
Sensory deficits	1 (7.1)	Progressive improvement with clinically insignificant residual symptoms

Percentages for full recovery, partial recovery, and death were calculated using the total cohort as the denominator (N = 46)

Percentages for residual neurological manifestations were calculated using patients with partial recovery as the denominator (n = 14)

Additional follow-up beyond the predefined study endpoint was available for all 14 patients with partial recovery. The most frequent residual manifestations at the 1-month assessment included residual seizures (*n* = 3), mild motor deficits including hemiparesis (*n* = 2), cognitive impairment (*n* = 2), developmental delay or behavioral abnormalities (*n* = 2), persistent headache or concentration difficulties (*n* = 3), and sensory deficits (*n* = 1).

Serial neurological evaluations demonstrated objective clinical improvement in all patients during subsequent follow-up. Among patients with residual seizures, seizure frequency progressively decreased, and complete seizure control was achieved in one patient. Patients with motor deficits exhibited substantial functional recovery, with only minimal residual weakness persisting in a minority of cases at the latest clinical evaluation. Similarly, patients with cognitive impairment, developmental delay, concentration difficulties, or behavioral abnormalities demonstrated gradual neurological and functional improvement during serial follow-up assessments.

Persistent headache and sensory deficits also improved over time, with symptoms either resolving completely or becoming clinically insignificant. Several patients achieved complete resolution of all residual neurological manifestations during follow-up, whereas the remaining patients continued to improve and exhibited only minor residual neurological deficits at their latest clinical evaluation. Importantly, no patient experienced neurological deterioration, recurrent hospitalization related to the index neurological illness, or death during the available follow-up period.

Overall, neurological recovery continued beyond the predefined 1-month study endpoint in patients initially classified as having partial recovery, indicating that early residual neurological deficits did not necessarily predict an unfavorable longer-term clinical outcome.

### Detection and molecular characterization of MCPyV

MCPyV DNA was detected in the CSF of 6 of 46 pediatric patients (13.0%) using TaqMan probe-based qPCR targeting the LT-ag gene, with all positive specimens subsequently confirmed by amplification of the independent VP1 target. A cycle threshold (Ct) value ≤ 38 was considered positive. All qPCR-positive samples underwent confirmatory bidirectional Sanger sequencing following re-amplification by a separate conventional PCR assay targeting the LT-ag region. Sequence analysis demonstrated 99.1–100% nucleotide identity with reference MCPyV strains available in the GenBank database. No SV40 DNA was detected in any CSF specimen.

**Fig. 1:**
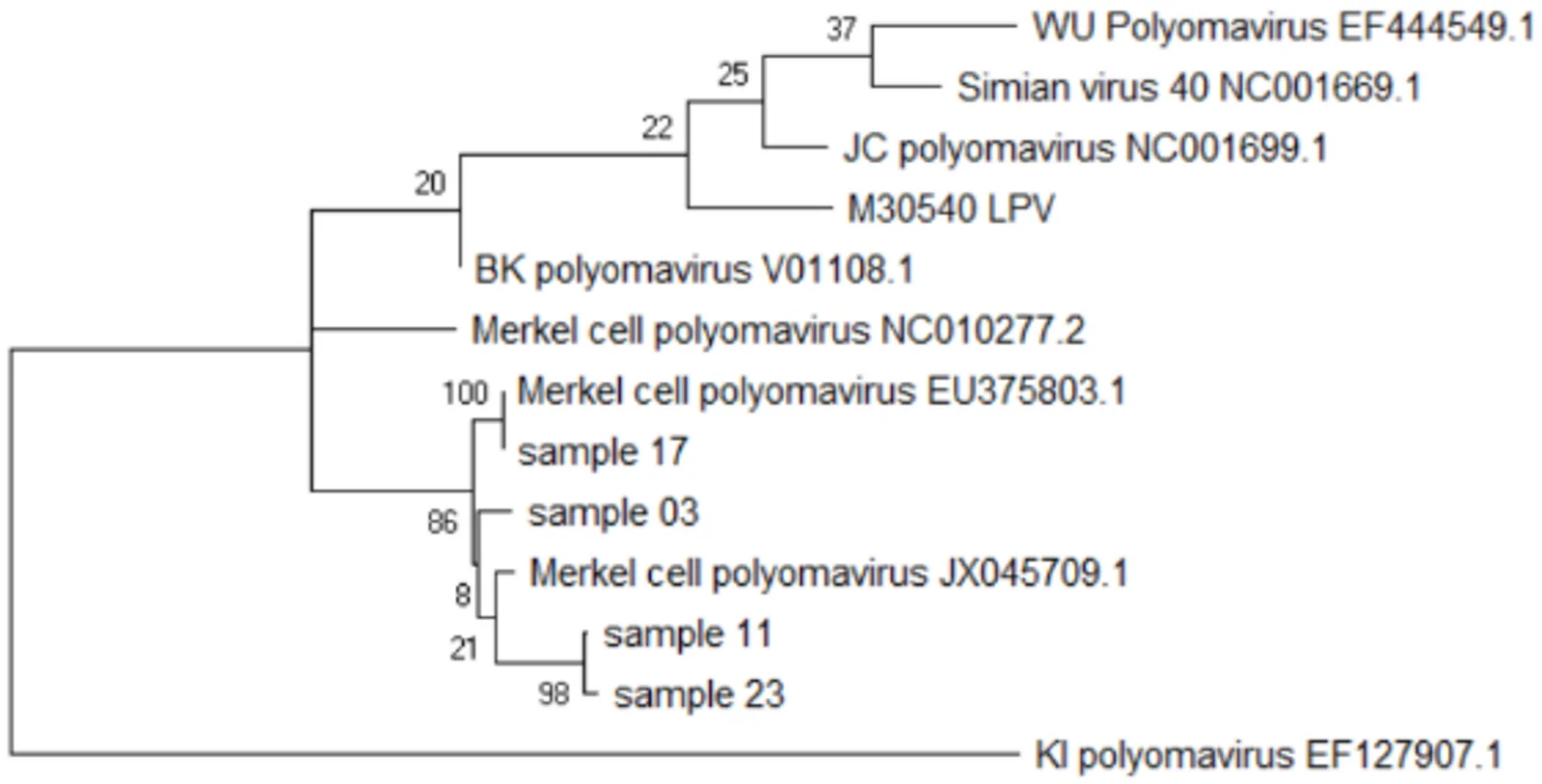
Phylogenetic analysis of MCPyV isolates

Among MCPyV-positive patients, the median Ct value was 32.5 (IQR: 31.5–33.8). Lower Ct values were interpreted as indicating higher MCPyV DNA burden. All qPCR assays included appropriate positive controls (MCPyV-positive Merkel cell carcinoma lysate) and negative controls (nuclease-free water and laboratory-negative CSF specimens). All positive samples were confirmed by amplification of the independent VP1 target and by bidirectional Sanger sequencing. Phylogenetic analysis of the LT-ag region demonstrated that four isolates (66.7%) clustered with MCPyV Genotype I, whereas two isolates (33.3%) clustered with Genotype IIc (Fig. 1). Sequence alignment was performed using the ClustalW algorithm implemented in MEGA software (version 7.0), and phylogenetic trees were constructed using the neighbor-joining method with the Kimura two-parameter substitution model. Tree topology was evaluated by bootstrap analysis with 1,000 replicates and visualized using FigTree software (version 1.4.4). Among the MCPyV-positive patients, one patient (P-17) had concurrent EBV DNA detection, representing 16.7% of MCPyV-positive cases. All other viral co-pathogens tested, including HSV-1/2, VZV, HHV-6, and enteroviruses, were negative, supporting the specificity of MCPyV detection in CSF (Table 4).

**Table 4:** Molecular and virological characteristics of MCPyV-positive patients (*n* = 6)

Patient Code	LT-ag Ct Value	MCPyV Genotype	Other Viruses in CSF	Sequencing Homology (%)
P-03	32.1	Type I	None	99.7
P-11	33.8	Type IIc	None	99.4
P-17	31.5	Type I	EBV DNA	100
P-23	34.5	Type IIc	None	99.1
P-35	30.8	Type I	None	99.8
P-41	32.8	Type I	None	99.6
Median (IQR)	32.5 (31.5–33.8)			

MCPyV DNA was detected using TaqMan probe-based qPCR targeting the LT-ag gene and confirmed by amplification of the independent VP1 target

All positive specimens were further confirmed by bidirectional Sanger sequencing following conventional PCR re-amplification of the LT-ag region

Ct, cycle threshold; CSF, cerebrospinal fluid; EBV, Epstein–Barr virus; IQR, interquartile range; MCPyV, Merkel cell polyomavirus

### Association of MCPyV positivity with HIV status

All six MCPyV-positive patients (100%) were HIV-positive, whereas no MCPyV DNA was detected in HIV-negative or HIV-unknown patients. Fisher’s exact test revealed a statistically significant association between MCPyV positivity and HIV infection (*p* = 0.001; Table 5). A sensitivity analysis excluding patients with unknown HIV status (*n* = 32) confirmed this association (*p* = 0.002; Table 6). Cramer’s V value of 0.543 indicated a strong association between HIV seropositivity and MCPyV detection in CSF.

The six MCPyV-positive patients exhibited a median Ct value of 32.5 (IQR: 31.5–33.8). To further explore the clinical significance of MCPyV detection, HIV-positive patients were stratified into those with MCPyV detection (HIV+/MCPyV+, *n* = 6) and those without MCPyV detection (HIV+/MCPyV−, *n* = 11), and comparisons were conducted across demographic, clinical, CSF, and outcome parameters.

Demographically, HIV+/MCPyV+ patients had a mean age of 6.8 ± 3.5 years, compared with 6.1 ± 3.9 years in HIV+/MCPyV− patients, with balanced gender distribution between groups.

CSF analysis revealed median WBC counts of 102 cells/µL (IQR: 88–128) and protein concentrations of 75 mg/dL (IQR: 68–82) in HIV+/MCPyV+ patients, compared with 78 cells/µL (IQR: 60–105) and 68 mg/dL (IQR: 60–85) in HIV+/MCPyV− patients. Median glucose concentrations were similar between groups (HIV+/MCPyV+: 55 mg/dL [IQR: 51–58]; HIV+/MCPyV−: 53 mg/dL [IQR: 49–58]).

Clinically, seizures occurred in 50% of HIV+/MCPyV+ patients compared with 36% of HIV+/MCPyV− patients. Altered consciousness was observed in 50% and 45% of patients, respectively, and fever was present in all patients in both groups. No deaths occurred.

Outcome analysis demonstrated that full recovery was achieved in 2/6 HIV+/MCPyV+ patients (33.3%) and 8/11 HIV+/MCPyV− patients (72.7%), whereas partial recovery occurred in 4/6 (66.7%) and 3/11 (27.3%) patients, respectively (Table 7).

Statistical testing using the Mann–Whitney U test for continuous variables and Fisher’s exact test for categorical variables revealed exploratory trends toward higher CSF WBC count and protein concentration, increased seizure frequency, and higher rates of partial recovery in HIV+/MCPyV+ patients. Formal statistical significance was limited by the small sample size.

Spearman’s rank correlation indicated a moderate inverse relationship between Ct values and full recovery (*r* = − 0.52), suggesting that higher MCPyV DNA burden, reflected by lower Ct values, may be associated with incomplete neurological recovery. These findings should be interpreted cautiously because of the exploratory nature of the analysis and the small number of MCPyV-positive cases.

**Table 5:** Contingency table of MCPyV positivity by HIV status

	HIV Positive	HIV Negative	HIV Unknown	Total
MCPyV Positive	6	0	0	6
MCPyV Negative	11	15	14	40
Total	17	15	14	46

Fisher's exact test (3 × 2): p = 0.001; Cramer's V = 0.543

**Table 6:** Sensitivity analysis excluding patients with unknown HIV status (*n* = 32)

	HIV Positive	HIV Negative	Total
MCPyV Positive	6	0	6
MCPyV Negative	11	15	26
Total	17	15	32

Fisher's exact test (2 × 2): p = 0.002

**Table 7:** Comparative summary of HIV+/MCPyV + and HIV+/MCPyV− patients

Parameter	HIV+/MCPyV+ (*n* = 6)	HIV+/MCPyV- (*n* = 11)	*p*-value
Age (years), mean ± SD	6.8 ± 3.5	6.1 ± 3.9	0.67
Gender, n (%) Male	3 (50%)	6 (54.5%)	1.0
CSF WBC, median (IQR)	102 (88–128)	78 (60–105)	0.12
CSF Protein, median (IQR)	75 (68–82)	68 (60–85)	0.31
CSF Glucose, median (IQR)	55 (51–58)	53 (49–58)	0.48
Seizure, n (%)	3 (50%)	4 (36%)	0.63
Altered consciousness, n (%)	3 (50%)	5 (45%)	1.0
ICU admission, n (%)	1 (16.7%)	1 (9.1%)	1.0
Full Recovery, n (%)	2 (33.3%)	8 (72.7%)	0.12
Partial Recovery, n (%)	4 (66.7%)	3 (27.3%)	0.12
Death, n (%)	0	0	–

### Comparison of clinical and CSF Parameters between MCPyV-positive and MCPyV-negative groups

To assess whether MCPyV detection in CSF was associated with differences in clinical presentation or CSF parameters, MCPyV-positive patients (*n* = 6) were compared with MCPyV-negative patients (*n* = 40).

The Shapiro–Wilk test demonstrated that CSF parameters, including WBC count, protein concentration, and glucose concentration, had a non-normal distribution (*p* < 0.001 for all), whereas age was normally distributed (*p* = 0.134). Accordingly, non-parametric tests were used for CSF parameter comparisons, and the independent-samples t-test was used for age comparison.

**Fig. 2:**
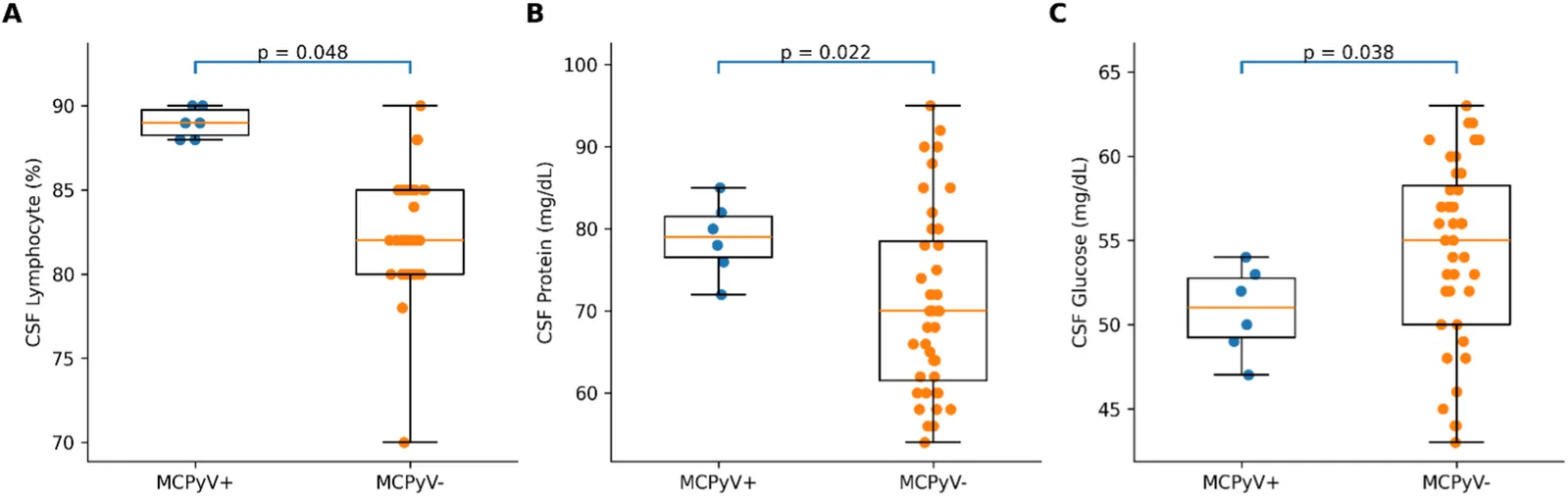
Comparison of CSF parameters between MCPyV-positive and MCPyV-negative groups. Box plots show (**A**) CSF lymphocyte percentage, (**B**) CSF protein concentration, and (**C**) CSF glucose concentration. MCPyV-positive patients (*n* = 6) had significantly higher lymphocyte percentages (*p* = 0.048), higher protein concentrations (*p* = 0.022), and lower glucose concentrations (*p* = 0.038) compared with MCPyV-negative patients (*n* = 40). Boxes represent the interquartile range (IQR), horizontal lines represent the median, and whiskers represent the range

MCPyV-positive patients exhibited significantly higher CSF lymphocyte percentages (median: 89% vs. 84%; *p* = 0.048), higher CSF protein concentrations (median: 79 vs. 68 mg/dL; *p* = 0.022), and lower CSF glucose concentrations (median: 51 vs. 55 mg/dL; *p* = 0.038) compared with MCPyV-negative patients (Fig. 2). Regarding clinical manifestations, MCPyV-positive patients had a significantly higher frequency of altered consciousness (83.3% vs. 37.5%; *p* = 0.039). Seizures were also more frequent among MCPyV-positive patients, although this difference did not reach statistical significance (66.7% vs. 30.0%; *p* = 0.082). No significant differences were observed in age (*p* = 0.191), gender (*p* = 0.380), neck stiffness (*p* = 0.554), or full recovery (*p* = 1.000).

Quantitative comparisons of demographic, clinical, and CSF parameters between MCPyV-positive and MCPyV-negative patients are summarized in Table 8. Interpretation of these comparisons is limited by the small number of MCPyV-positive cases and the exploratory nature of the analysis.

**Table 8:** Comparison of demographic, clinical, and CSF parameters between MCPyV-positive and MCPyV-negative groups

Parameter	MCPyV Positive (*n* = 6)	MCPyV Negative (*n* = 40)	Test statistic	*P*-value
Age (years), mean ± SD	7.5 ± 3.2	6.2 ± 3.9	t = 1.33	0.191
Gender, Male n (%)	4 (66.7)	19 (47.5)	χ² = 0.77	0.380
HIV Positive, n (%)	6 (100)	11 (27.5)	Fisher’s Exact	0.001*
CSF WBC (cells/µL), median (IQR)	103 (98–110)	82 (60–125)	U = 86.5	0.216
CSF Lymphocytes (%), median (IQR)	89 (88–90)	84 (82–88)	U = 74.5	0.048*
CSF Protein (mg/dL), median (IQR)	79 (75–82)	68 (60–80)	U = 68.0	0.022*
CSF Glucose (mg/dL), median (IQR)	51 (49–53)	55 (50–58)	U = 72.5	0.038*
Seizure, n (%)	4 (66.7%)	12 (30.0%)	Fisher’s Exact	0.082
Altered consciousness, n (%)	5 (83.3%)	15 (37.5%)	Fisher’s Exact	0.039*
Neck stiffness, n (%)	3 (50.0%)	25 (62.5%)	χ² = 0.35	0.554
Full recovery, n (%)	4 (66.7%)	28 (70.0%)	Fisher’s Exact	1.000

CSF, cerebrospinal fluid; IQR, interquartile range; SD, standard deviation; WBC, white blood cell count

### Correlation analysis between viral load and CSF parameters

To investigate whether MCPyV DNA burden was associated with the magnitude of the CSF inflammatory response, correlations between Ct values and CSF WBC count, protein concentration, and glucose concentration were assessed among MCPyV-positive patients (*n* = 6). Ct values were used as the quantitative indicator of MCPyV DNA burden, with lower Ct values indicating higher MCPyV DNA burden.

Moderate negative correlations were observed between Ct value and CSF WBC count (Spearman’s rho = − 0.54, *p* = 0.267) and between Ct value and CSF protein concentration (Spearman’s rho = − 0.61, *p* = 0.200). These negative correlations suggest that higher MCPyV DNA burden, reflected by lower Ct values, tended to be associated with more pronounced CSF pleocytosis and protein elevation. Conversely, a moderate positive correlation was observed between Ct value and CSF glucose concentration (Spearman’s rho = + 0.48, *p* = 0.338), indicating that lower MCPyV DNA burden, reflected by higher Ct values, tended to be associated with higher CSF glucose concentrations.

**Fig. 3:**
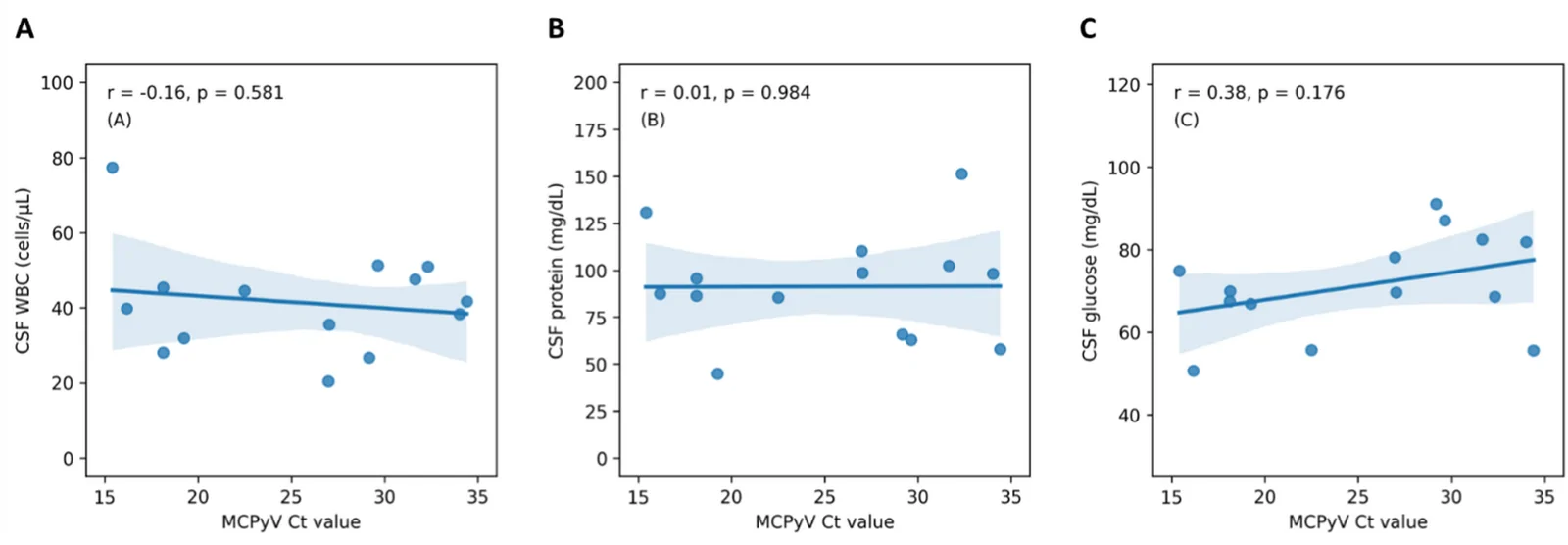
Scatter plots showing correlations between MCPyV Ct values and CSF parameters among MCPyV-positive patients. (**A**) Ct value versus CSF WBC count (rho = − 0.54, *p* = 0.267), (**B**) Ct value versus CSF protein concentration (rho = − 0.61, *p* = 0.200), and (**C**) Ct value versus CSF glucose concentration (rho = + 0.48, *p* = 0.338). Lower Ct values indicate higher MCPyV DNA burden. None of the correlations reached statistical significance

None of these correlations reached statistical significance, likely because of the limited number of MCPyV-positive cases. Therefore, these findings should be interpreted as exploratory and hypothesis-generating rather than confirmatory (Fig. 3).

### Summary of main findings

The overall detection rate of MCPyV DNA in CSF samples from pediatric patients with suspected viral encephalitis or meningoencephalitis was 13.0% (6/46), as determined by TaqMan probe-based qPCR and confirmed by bidirectional Sanger sequencing. All six MCPyV-positive patients were HIV-positive, whereas no MCPyV DNA was detected in HIV-negative or HIV-unknown patients. This association remained statistically significant in the full cohort analysis (Fisher’s exact test, *p* = 0.001; Cramer’s V = 0.543) and in the sensitivity analysis excluding patients with unknown HIV status (*p* = 0.002).

Molecular characterization showed that four MCPyV isolates clustered with Genotype I and two with Genotype IIc. Among MCPyV-positive patients, the median Ct value was 32.5 (IQR: 31.5–33.8). Systematic screening for common viral co-pathogens identified EBV DNA co-detection in one MCPyV-positive patient, whereas HSV-1/2, VZV, HHV-6, and enteroviruses were not detected in MCPyV-positive cases.

Comparison of MCPyV-positive and MCPyV-negative patients showed significantly higher CSF lymphocyte percentages, higher CSF protein concentrations, and lower CSF glucose concentrations in MCPyV-positive patients. Altered consciousness was also more frequent in the MCPyV-positive group. Subgroup comparison among HIV-positive patients showed exploratory trends toward higher CSF WBC count and protein concentration, more frequent seizures, and higher partial recovery rates in HIV+/MCPyV+ patients compared with HIV+/MCPyV− patients. However, these findings should be interpreted cautiously because of the small number of MCPyV-positive cases. Spearman correlation analysis also suggested non-significant trends between lower Ct values and greater CSF inflammatory activity, supporting the need for larger prospective studies.

## Discussion

In this study, we investigated the presence of MCPyV DNA in the CSF of 46 paediatric patients with suspected viral encephalitis or meningoencephalitis. MCPyV DNA was detected in 13.0% of cases and was observed exclusively among HIV-positive children. This finding indicates a strong association between MCPyV detection in CSF and HIV-positive status in this cohort; however, it should not be interpreted as evidence of causality. MCPyV-positive patients also showed higher CSF lymphocyte percentages, higher protein levels, lower glucose levels, and a higher frequency of altered consciousness compared with MCPyV-negative patients. Taken together, these findings suggest that MCPyV detection in CSF may represent a clinically relevant virological signal in immunocompromised paediatric patients, but the data remain hypothesis-generating rather than definitive evidence of MCPyV-associated CNS disease [39–42].

The overall detection rate of MCPyV DNA in our cohort was broadly comparable to previous reports of MCPyV detection in CSF. Delbue et al. detected MCPyV DNA in 9.4% of CSF samples from patients with heterogeneous neurological disorders, including meningoencephalitis, neuropathy, migraine, multiple sclerosis, and brain tumors [1]. The slightly higher detection rate in the present study may reflect differences in patient selection, as our cohort consisted exclusively of children investigated for suspected viral CNS infection, whereas prior studies included broader neurological populations. Geographic and age-related differences in MCPyV exposure may also contribute [30, 43, 44]. Previous seroepidemiological studies reported age-dependent MCPyV seroprevalence, with lower rates in younger children and increasing seropositivity during adolescence and adulthood [45]. This pattern is consistent with reports from other populations showing that MCPyV infection is commonly acquired during childhood, probably through close interpersonal contact involving skin or saliva [19, 46, 47].

A central observation of this study was the exclusive detection of MCPyV DNA among HIV-positive children. This association is biologically plausible because impaired cellular immunity may facilitate polyomavirus persistence, reactivation, or dissemination [34, 48–57]. Nevertheless, HIV positivity alone is not equivalent to severe immunosuppression, and interpretation requires clinical immunological context, including CD4 count, CD8 count, HIV viral load, ART status, duration of infection, and AIDS stage [58]. In our study, HIV-positive patients were therefore evaluated as an immunocompromised subgroup, and comparisons were performed between HIV+/MCPyV + and HIV+/MCPyV− patients. This subgroup analysis was added specifically to distinguish the effect of MCPyV detection from HIV status itself, although the small number of MCPyV-positive cases limits statistical power. The findings are consistent with the broader behavior of human polyomaviruses, particularly JCPyV and BKPyV, which can reactivate under impaired immune surveillance [3, 51]. However, unlike JCPyV-associated progressive multifocal leukoencephalopathy, the neuropathogenic role of MCPyV remains unproven [7, 55, 59–65].

The systematic assessment of viral co-pathogens is important for interpreting MCPyV detection in CSF. In our study design, all CSF samples were screened for common viral CNS pathogens, including HSV-1/2, VZV, EBV, HHV-6, and enteroviruses. Only one MCPyV-positive patient had EBV DNA co-detection, while the remaining MCPyV-positive cases were negative for the tested viral co-pathogens. This strengthens the relevance of MCPyV detection, but it still does not prove that MCPyV was the causal agent of disease. EBV co-detection in one patient also underscores the need to interpret positive CSF viral PCR results within the broader clinical, immunological, and virological context, particularly in HIV-infected children.

The CSF profile of MCPyV-positive patients showed higher lymphocyte percentages, elevated protein levels, and lower glucose levels compared with MCPyV-negative patients. These findings are compatible with inflammatory CNS involvement but are not specific for MCPyV infection. Similar CSF abnormalities may occur in viral CNS infections, HIV-associated CNS disease, immune activation, or opportunistic co-infections. The higher frequency of altered consciousness among MCPyV-positive patients may indicate greater neurological severity, but the observational design does not allow causal attribution. Therefore, MCPyV should be interpreted as a possible contributing or reactivation-associated marker rather than a confirmed primary neurotropic pathogen.

The use of TaqMan probe-based qPCR allowed detection of MCPyV DNA, with Ct values used as indicators of MCPyV DNA burden. Lower Ct values tended to correlate with higher CSF WBC and protein concentrations and lower glucose concentrations, although these associations were not statistically significant. These non-significant trends may suggest a relationship between MCPyV DNA burden and CSF inflammatory activity, but the small number of positive cases precludes firm inference. Larger prospective studies using standardized TaqMan probe-based quantitative PCR assays with harmonized viral load reporting, paired CSF and blood testing, and longitudinal sampling are required to determine whether MCPyV DNA burden correlates with neurological severity or outcome.

Phylogenetic analysis showed that four isolates clustered with MCPyV Genotype I and two with Genotype IIc. Genotype I is widely reported in different populations, whereas Genotype IIc has been more frequently described in East Asian settings [44, 66].

The detection of both genotypes in this Iranian pediatric cohort may reflect regional viral diversity, population movement, or broader-than-recognized circulation of MCPyV genotypes. However, no conclusion can be drawn regarding genotype-specific neurotropism or virulence. Whole-genome sequencing and larger geographically diverse datasets will be needed to determine whether particular MCPyV variants are associated with CNS detection, immunosuppression, or neurological severity.

Partial recovery was defined using predefined criteria, including persistent neurological deficits, residual seizures, cognitive or developmental impairment, or hospitalization exceeding 14 days. In subgroup analysis, HIV+/MCPyV+ patients showed exploratory trends toward higher partial recovery rates compared with HIV+/MCPyV− patients, although these differences were not statistically significant. MCPyV detection in CSF may be clinically informative in selected immunocompromised children, but there is insufficient evidence to recommend routine MCPyV testing as a stand-alone diagnostic or prognostic tool. Its potential value may be greatest when common viral pathogens are negative and the patient has advanced immunosuppression.

Several limitations should be acknowledged. First, the sample size was small, particularly the MCPyV-positive subgroup, limiting statistical power. Second, HIV status was unavailable for a subset of patients, although sensitivity analysis excluding unknown HIV status confirmed the association between MCPyV detection and HIV positivity. Third, although HIV-related immunological variables were collected and incorporated into the analysis, the small number of HIV-positive/MCPyV-positive patients limited the ability to stratify outcomes according to the degree of immunosuppression, ART duration, or HIV disease severity. Fourth, MCPyV serology was not performed; therefore, we could not determine whether CSF detection reflected primary infection, reactivation in previously seropositive children, or incidental DNA detection. Fifth, the absence of a non-CNS control group limits interpretation of background MCPyV DNA detection in CSF. Sixth, detection of MCPyV DNA in CSF does not establish causality and may reflect latent viral persistence, reactivation, passive transfer from blood, or contamination from skin during lumbar puncture. Seventh, comprehensive neuroimaging and long-term neurodevelopmental follow-up were not available for all patients. Finally, the single-center design may limit generalizability.

In conclusion, this study identifies MCPyV DNA in the CSF of a subset of children with suspected viral CNS infection, with detection restricted to HIV-positive patients. The findings support a possible association between MCPyV detection, immunosuppression, and inflammatory CSF abnormalities, but they do not prove that MCPyV is a causal agent of pediatric meningitis or encephalitis. Future multicenter prospective studies should include standardized TaqMan probe-based qPCR assays with harmonized Ct value reporting, paired CSF and blood testing, MCPyV serology, systematic co-pathogen panels, non-CNS control groups, neuroimaging, and longitudinal neurological follow-up to clarify the clinical significance of MCPyV detection in pediatric CSF.

## Conclusion

This study demonstrates the detection of MCPyV DNA in the CSF of a subset of HIV-positive pediatric patients with suspected viral meningoencephalitis in Iran. MCPyV detection was exclusively observed among HIV-positive children and was associated with inflammatory CSF abnormalities, including higher lymphocyte percentages, elevated protein levels, lower glucose concentrations, and a higher frequency of altered consciousness. These findings support a possible association between MCPyV detection, immunosuppression, and CNS inflammatory involvement, but they do not establish MCPyV as a definitive causal pathogen of pediatric meningitis or encephalitis.

The systematic exclusion of common viral co-pathogens in most MCPyV-positive cases increases the potential clinical relevance of these findings, particularly in immunocompromised pediatric patients with negative routine viral investigations. Accordingly, MCPyV may warrant consideration as a possible opportunistic or reactivation-associated viral marker in selected HIV-infected children presenting with suspected CNS infection. However, the clinical significance of MCPyV detection in CSF remains incompletely understood.

Future multicenter prospective studies with larger cohorts are required to clarify the neuropathogenic potential of MCPyV. These investigations should incorporate standardized TaqMan probe-based qPCR assays with harmonized reporting of Ct values and, where available, absolute MCPyV DNA copy numbers, paired CSF and blood analyses, MCPyV serology, comprehensive co-pathogen screening, neuroimaging correlation, and longitudinal neurological follow-up. The inclusion of non-CNS control populations will also be essential to determine whether MCPyV detection in CSF reflects active neuroinvasive disease, viral reactivation, latent persistence, or incidental viral DNA shedding.

## Data Availability

No datasets were generated or analysed during the current study.
